# The Effects of Plasma Treated Electrospun Nanofibrous Poly (ε-caprolactone) Scaffolds with Different Orientations on Mouse Embryonic Stem Cell Proliferation 

**Published:** 2014-10-04

**Authors:** Naghmeh Abbasi, Sara Soudi, Nasim Hayati-Roodbari, Masumeh Dodel, Masoud Soleimani

**Affiliations:** 1 Department of Biology, School of Basic Science, Science and Research Branch, Islamic Azad University, Tehran, Iran; 2 Department of Stem Cell Biology, Stem Cell Technology Research Center, Tehran, Iran; 3 Department of Nanotechnology and Tissue Engineering, Stem Cell Technology Research Center, Tehran, Iran; 4 Department of Textile Engineering, Amirkabir University of Technology, Tehran, Iran; 5 Department of Hematology, Faculty of Medical Sciences, Tarbiat Modares University, Tehran, Iran

**Keywords:** Embryonic Stem Cells, Nanofibers, Poly (ε-caprolactone), Surface Modification, Cell Proliferation

## Abstract

**Objective:**

Assessments of cell reactions such as motility, orientation and activation to the topography of the substratum will assist with the fabrication of a proper implantable scaffold for future tissue engineering applications.The current challenge is to analyze the orientation effect of elecrospun nanofibers of poly (ε-caprolactone) (PCL) on viability and proliferation of mouse embryonic stem cells (mESCs).

**Materials and Methods:**

In this experimental study, we used the electrospinning method to fabricate nanofibrous PCL scaffolds. Chemical and mechanical characterizations were specified by the contact angle and tensile test. O_2_plasma treatment was used to improve surface hydrophilicity. We used the 3-(4,5-dimethylthiazol-2-yl)-2,5-diphenyltetrazolium bromide (MTT) assay to evaluate mESCs adhesion and proliferation before and after surface modification. The influence of the orientation of the nanofibers on mESCs growth was evaluated by scanning electron microscopy (SEM). Statistical analysis was performed using one-way analysis of variance (ANOVA) With differences considered statistically significant at p≤0.05.

**Results:**

The results showed that plasma treatment improved the hydrophilic property of PCL scaffolds. MTT assay showed a significant increase in proliferation of mESCs on plasma treated PCL (p-PCL) scaffolds compared to non-treated PCL (p=0.05). However gelatin coated tissue culture plate (TCP) had a better effect in initial cell attachment after one day of cell seeding. There was more cell proliferation on day 3 in aligned plasma treated (AP) nanofibers compared to the TCP. SEM showed optical density of the cell colonies. Aligned nanofibrous scaffolds had larger colony sizes and spread more than random nanofibrous scaffolds.

**Conclusion:**

This study showed that plasma treating of scaffolds was a more suitable substrate for growth and cell attachment. In addition, aligned nanofibrous scaffolds highly supported the proliferation and spreading of mESCs when compared to random nanofibrous scaffolds and TCP.

## Introduction

Autologous cell transplantation is a powerful strategy for treatment of injured tissues. However it is difficult to obtain sufficient amounts of autologous cells, specially when a patient is aged or has severely diseased (1). Cell injection techniques may result in inappropriate localization, differentiation, or orientation of cells at the injection site and may not show significant increases in functional recovery. Therefore, the use of tissue scaffolds as substratum for in situ proliferation, structuring and organizing cell populations is preferred by researchers (2).

Nanofibrous scaffold produced by electrospinning mimics the extracellular matrix (ECM). This scaffold can be a suitable candidate for tissue engineering. There are numerous varieties of synthetic polymers whose mechanical properties and degradation rates can be tailored for special applications (3-5). Although poly (ε-caprolactone) (PCL) is an aliphatic, biodegradable, non-toxic polyester with good mechanical properties, its hydrophobicity and deficient biological activity are unfavorable for cell adhesion and proliferation (6, 7). One problem of nanofibrous scaffold produced by electrospinning is cell penetration into the scaffolds, thus surface modification can influence the interactions between the material and biosystem which result in improvements to the substrate’s biocompatibility (8).

Plasma treatment, according to previous studies, is one of the post-processing surface modification techniques adopted to modify nanofibrous scaffolds. This treatment introduces desired functional groups onto the substratum to enhance cell adhesion and support proliferation on a hydrophobic surface (9, 10).

It has been shown in previous studies that the phenomenon of contact guidance led to the cells guiding along the parallel axis of electrospun fibers and elongation relating to the topography of seeded substratum to produce amounts of oriented ECM (11). Borjigin et al. used PCL blended fiber membranes as templates to grow genetically modified HCT116-19 colon cancer cells. Their study showed that aligned PCL nanofibers exhibited a 2.5-fold higher cell proliferation rate compared to cells plated on plastic plate surfaces (12). Wang et al. (13) analyzed the effect of aligned and randomly oriented electrospun collagen scaffolds on neural progenitor cells (NPCs). These researchers observed greater cell proliferation on the aligned nanofibers compared to the randomly oriented nanofibers and control.

The contact angle results of a study by Martins et al. showed decreased hydrophobicity of O_2_ plasmatreated electrospun PCL nanofiber meshes (14). In addition, an increase in hydrophilicity was reported by Shabani et al. after plasma treatment of nanofibrous polyethersulfone (PES) scaffolds (15).

Among candidates, embryonic stem cells (ESCs) have higher proliferation and differentiation potentials than mature cells. Therefore, they can provide an ideal cell source for tissue engineering (16).

There are no studies about the effects of topographical cues of oriented PCL nanofibers on proliferation of mouse ESCs (mESCs) that have compared three groups-aligned electrospun scaffolds, random electrospun scaffolds, and gelatin coated tissue culture plates (TCP). Therefore, the objective of this study is to evaluate the efficacy of plasma treated and untreated oriented nanofibrous PCL scaffolds in their ability to support the growth, proliferation and attachment of mESCs compared to gelatin coated TCP. This study also seeks to analyze the orientation of fibers on cell proliferation.

## Materials and Methods

### Fabrication of aligned and random nanofibrous scaffolds

In this experimental study, the PCL polymer (8% weight) solution in chloroform/DMF (Sigma-Aldrich, St. Louis, MO, USA) at the concentration of 1: 9, was loaded into a 5 ml plastic syringe. This liquid was extruded from the 21 gauge needle at a flow rate of 0.5 ml/hour by a syringe pump. The positive output lead of a high voltage supply was set to 25 kV and attached to a blunt 21 gauge needle on the syringe. A grounded target (aluminum steel) was placed 23 cm from the needle tip, at a revolution of 100 RPM for random fibrous mats and 1000 RPM for aligned fibrous mats. With the application of high voltage, the ejected solution formed a Taylor cone at the needle tip and was drawn toward the collector as a whipping jet. During the jet’s travel, the solvent gradually evaporated leaving a continuous polymer fiber that accumulated on the aluminum diameter. The fiber was allowed to dry overnight under vacuum prior to its use in the experiments.

### Plasma surface modification of electrospun nanofibers

For O_2_ plasma exposure of electrospun PCL nanofibrous scaffolds, we used Diener electronic plasma cleaner (Germany). Nanofibers were placed in the chamber of the plasma cleaner. An RF power of 30 W and exposure time of 3 minutes under vacuum mode was applied.

### Characterization of scaffolds

We studied the morphology of these nanofibrous scaffolds by scanning electron microscopy (SEM, Camscan mz2300, Czech Republic) at an accelerating voltage of 10 kV. The scaffolds were coated with gold by using a sputter coater (BIO-RAD Polaran e5400 Sputter Coater, UK). Then the diameter of the fiber was measured from the SEM micrographs by image analysis software VEGA TS 5136MM (School of Metallurgy and Material Engineering, Tehran University, Iran).

For determination of wettability or hydrophilicity of the PCL nanofibrous scaffolds before and after plasma treatment, the contact angle of electrospun nanofibers was measured by the sessile drop method and contact angle measuring system (OCA15plus, Germany) mounted with a chargecoupled device (CCD) camera. The droplet size was set at 0.5 ml. We used five samples for each test. The average result as the water contact angle was reported.

Mechanical properties of different scaffolds were determined using a tensile tester (SANTAM Stress Machine STM20, Iran) at a cross-head speed of 50 mm/minute under ambient conditions. All samples were prepared in the form of a rectangular shape (60×10 mm^2^) from the electrospun fibrous membranes. The ends of the rectangular specimens were mounted vertically on mechanical gripping units of the tensile tester. At least five samples were tested for each type of electrospun fibrous membrane. The obtained results were plotted to obtain the stress-strain curve for scaffolds.

To obtain the porosity of scaffolds, the apparent density of cutting electrospun scaffolds according to their mass and volume were measured. The length, width and thickness were measured by a micrometer. The rate of porosity was calculated according to the following equation:

$$\mathrm{P(\%)}=\left(\frac{1-\rho }{\rho_{̥}}\right)\times 100$$

$$\mathrm{\rho (g/cm³)}=\frac{m}{V}$$

where: P(%): porosity ; ρ (g/cm³): apparent density; ρ̥ (g/cm³): bulk density of the membranes; m(g): mass of the nanofiber membrane; V(cm³): volume. The bulk density of PCL was 1.146 g/cm^3^.

### In vitro culture of mouse embryonic stem cells (mESCs)

We sterilized the nanofibrous scaffolds in 70% ethanol for 2 hours after which they were washed 3 times with PBS for 20 minutes and subsequently incubated with DMEM/F12 (Sigma-Aldrich, MO, USA) for 24 hours before cell seeding. Mouse embryonic fibroblast (MEF) cells were prepared according to guidelines the Laboratory Animal Ethical Commission of Tarbiat Modares University and cultured in DMEM F12 supplemented with 10% FBS (Sigma-Aldrich, MO, USA). After reaching 70% confluency, the cells were chemically inactivated with mitomycin C (10 μg/ml; Gibco- Invitrogen, CA, USA) for 2 hours and washed 3 times with PBS to remove any remaining mitomycin C. mESCs, obtained from the Stem Cell Technology Research Center (17), were grown on a gelatinized plate that contained one layer of inactive feeder cells (MEFs) in ES culture medium that included Knock-Out DMEM (Sigma-Aldrich, MO, USA) which consisted of sodium pyruvate supplemented with 20% ESC qualified fetal bovine serum (Gibco-Invitrogen, CA, USA), 1000 IU/ml LIF (Gibco-Invitrogen, CA, USA), 1 mM NEAA, 2 mM L-glutamine, 0.1 mM β-ME, and 100 μg/ml pen/strep antibiotics (Gibco-Invitrogen, CA, USA). Cells were maintained in a humidified CO_2_ incubator at 37˚C. Media was changed daily until the appropriate confluency of ESC colonies was attained.

### MTT assay for mESC proliferation

After mESCs were dissociated from the feeder cells we subsequently seeded them onto the following: i. random plasma treated PCL (Rp-PCL) nanofibrous scaffolds, ii. aligned plasma treated PCL (Ap-PCL) nanofibrous scaffolds, iii. untreated random PCL (R-PCL) nanofibrous scaffolds, iv. aligned PCL (A-PCL) nanofibrous scaffolds, and v. tissue culture plates (TCP) as the control, in 96-well plates at a density of 10×10^3^ cells/well. We monitored cell viability and proliferation on the different substrates after 1, 3 and 5 days of cell seeding with the colorimetric 3-(4,5-dimethylthiazol-2-yl)-2,5-diphenyltetrazolium bromide (MTT) assay (18). Following the designated time periods, the cell constructs were rinsed with phosphate-buffered saline (PBS) and incubated with 10% MTT reagent (a yellow tetrazole) that contained serum-free medium. After 4 hours of incubation at 37˚C in 5% CO_2_ the aliquots were pipetted. Cells that were metabolically active reacted to tetrazolium salt in MTT reagent to produce a soluble formazan dye. The absorbance of the content of each well was measured at 540 nm by a spectrophotometric plate reader (Eppendorf Bio Photometer, Germany). To release the formazan crystals from scaffolds, the electrospun nanofibers were dissolved in chloroform solution.

### Morphology of mESCs

We used SEM to study the morphology of mESCs on O_2_ plasma treated and untreated aligned and random nanofibrous PCL. After 3 days of cell seeding, samples were fixed with 2.5% glutaraldehyde for 3 hours. Samples were dehydrated in increasing concentrations (60, 70, 80, 90 and 100% v/v) of ethanol for 15 minutes each after which they were placed under a fume hood and allowed to air dry. Finally the scaffolds were coated with gold for observation of cell morphology by SEM.

### Statistical analysis

All data were obtained at least in triplicate, averaged, and expressed as mean ± standard deviation (SD). The experiment was repeated twice. We used one-way analysis of variance (ANOVA) for statistical analysis. Differences were considered statistically significant at p≤0.05.

## Results

### Characterization of scaffolds

**Fig 1:**
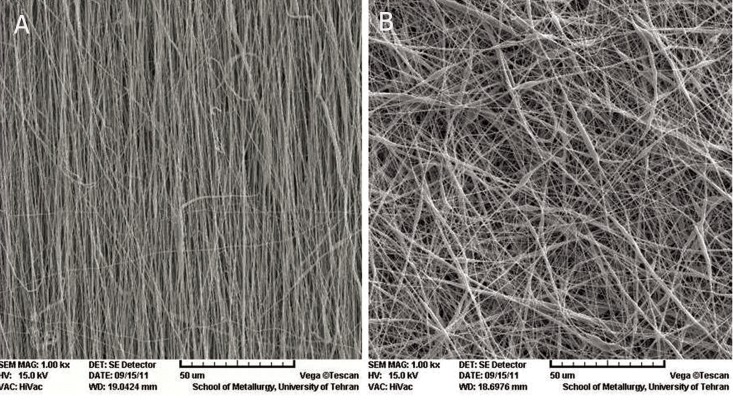
SEM micrograph of nanofiber: A. aligned poly (ε-caprolactone) (PCL) nanofiber and B. random PCL nanofiber.

SEM micrographs of the electrospun nanofibrous scaffolds revealed nanoscaled structures with interconnected pores that formed under controlled parameters. Randomly and aligned PCL nanofibers showed uniform thicknesses, bead-free nanofibers and interconnected pores with fiber diameters that ranged from 250-267 nm (Fig 1A, B). SEM images showed no changes in surface morphology of the PCL nanofiber after plasma treatment.

Measurements of the contact angle by water droplet showed surface hydrophilicity property. As seen in table 1, the contact angle obtained from the untreated A-PCL scaffold was 133 and for the R-PCL scaffold it was 134.5 which implied that they were highly hydrophobic and didn’t absorb the water. The plasma treated nanofibrous scaffolds (Ap-PCL and Rp-PCL) with zero water contact angle and rapid absorbance of the water droplet had an extremely hydrophilic property. This confirmed that the higher hydrophilic behavior of Ap-PCL and Rp-PCL was directly attributed to the presence of hydrophilic groups on the surface of the scaffold.

**Fig 2:**
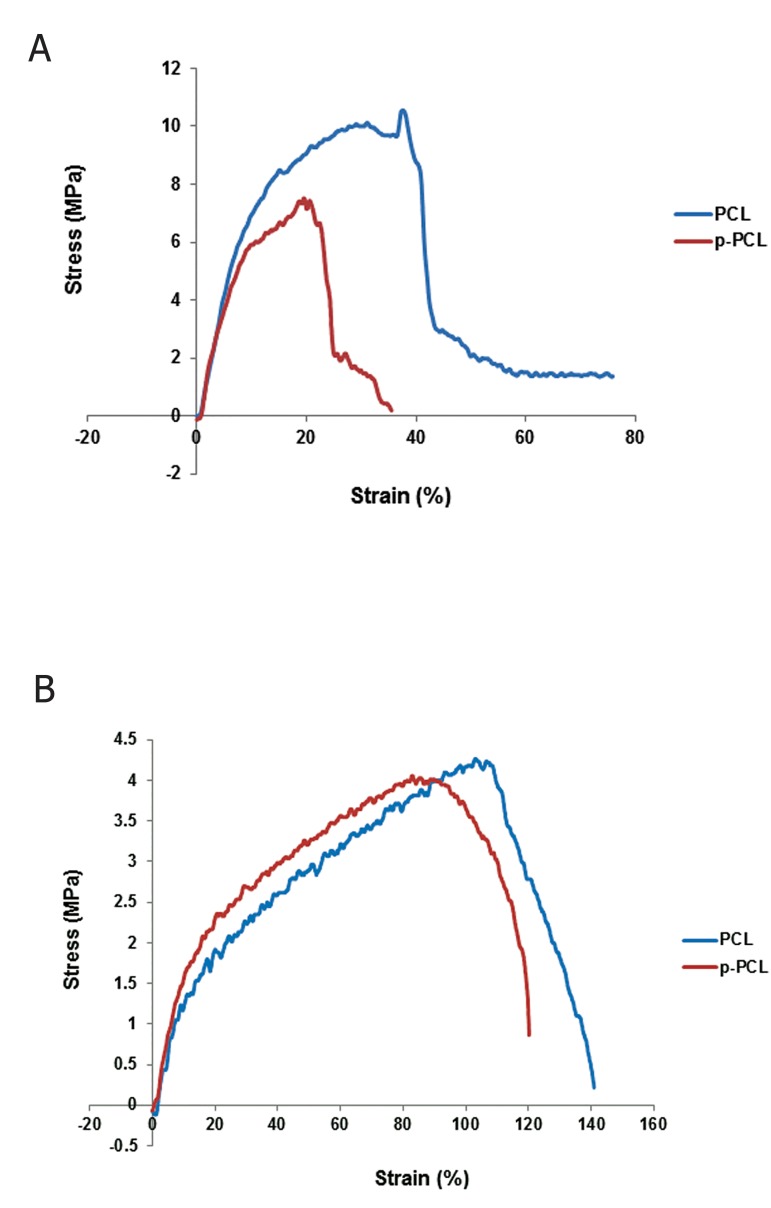
Stress-strain curve of poly (ε-caprolactone) (PCL) and plasma treated PCL (p-PCL) aligned (A) and random (B) nanofibers. MPa; Mega Pascal.

A stress testing machine was used to separately determine the electrospun nanofibers’ mechanical properties. Figure 2 shows the stressstrain curves of electrospun nanofibrous p-PCL and PCL oriented perpendicular (A) and parallel (B) to the rotating collector. Table 2 gives the mechanical parameters, such as Young’s modulus (mean E), peak stress, and peak strain of the four structures. The random and aligned p-PCL nanofibrous scaffolds showed reduced mechanical strength and elongation. According to one-way ANOVA there was a significant difference (p≤0.05) among the modulus, ultimate strength, break strain of the A-PCL, which had higher Young’s moduli and maximum break stress compared to the random PCL. However, the maximum strength at break in the aligned PCL was lower than the random PCL.

**Table 1:** Analyses of the aligned and random electrospun poly (ε-caprolactone) (PCL) and plasma treated PCL (p-PCL) nanofiber properties


Properties	Aligned PCL (A-PCL)	Aligned p-PCL(Ap-PCL)	Random PCL(R-PCL)	Random p-PCL(Rp-PCL)

**Porosity (%)**	70.3	70	77.4	77
**Contact angle (degree)**	133	0	135.5	0
**Wettability**	Highly hydrophobic	Highly hydrophilic	Highly hydrophobic	Highly hydrophilic


**Table 2:** Tensile properties of poly (ε-caprolactone) (PCL) nanofibers


Nanofiber scaffold	Tensile stress (MPa)	Tensile strain (%)	Elastic module (MPa)

**Random PCL (R-PCL)**	4.26	103.2	3.16
**Random plasma treated PCL (Rp-PCL)**	4.02	85.83	2.9
**Aligned PCL(A-PCL)**	10.11	31.05	18.27
**Aligned plasma treated PCL(Ap-PCL)**	7.42	20.67	18.06


### Cell proliferation

**Fig 3:**
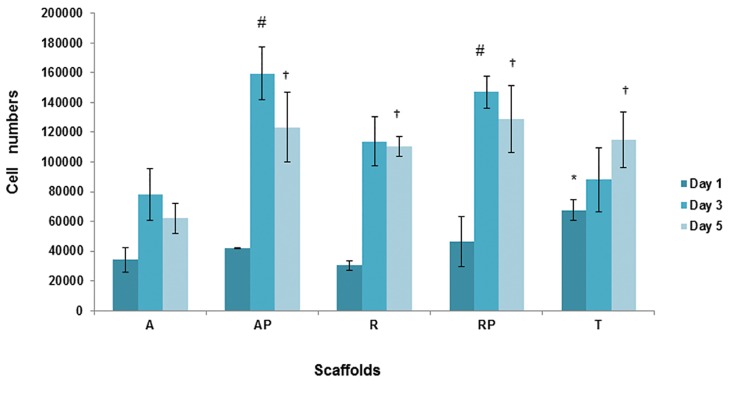
MTT assay for mouse embryonic stem cell (mESCs) proliferation on poly (ε-caprolactone) (PCL) and plasma treated PCL (p-PCL) aligned and random nanofiber scaffolds and gelatin coated tissue culture plate (TCP). Bar represents mean ± standard deviation. *; Significant level of adherance and proliferation on TCP compared to aligned PCL (A-PCL), random PCL (R-PCL), aligned plasma treated PCL (Ap-PCL), random plasma treated PCL (Rp-PCL) on day 1 at ≤0.05, #; Significant level of proliferation on plasma treated random and aligned nanofiber compared to untreated scaffolds and gelatin coated TCP on day 3 at ≤0.05,†; Significant reduction of proliferation level on aligned PCL scaffold compared to the other groups on day 5 at ≤0.05, OD; Optic density, AP; Plasma treated aligned nanofibrous scaffold, RP; Plasma treated random nanofibrous scaffold, A; Nontreated aligned nanofibrous scaffold, R; Nontreated random nanofibrous scaffold and T; Gelatin coated tissue culture plate (TCP).

Cell attachment and proliferation were measured by the MTT assay (Fig 3). A comparison of cell numbers on day one after cell culture showed the attachment potential of each matrix. According to Figure 3, TCP had a higher degree of initial cell attachment compared to plasma treated and nontreated random and aligned nanofibrous scaffolds. On day 3 after cell culture, we observed a significantly higher cell proliferation (p≤0.05) in the nontreated random scaffolds compared to the aligned scaffolds. The results showed that cell proliferation was induced significantly in both random and aligned plasma treated scaffolds. After 5 days of cell culture, the cell numbers were reduced in all groups, with the exception of the gelatin coated TCP.

### Morphological studies of mESCs

**Fig 4:**
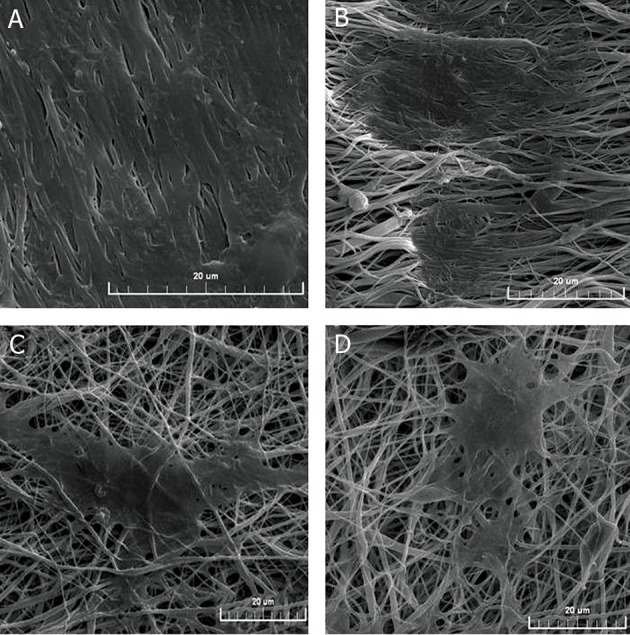
SEM micrograph of mouse embryonic stem cells (mESCs) on nanofibrous poly (ε-caprolactone) (PCL) scaffolds after 3 days of cell seeding: A. plasma treated aligned nanofibers (Ap-PCL), B. non-treated aligned nanofibers (A-PCL), C. plasma treated random nanofibers (Rp-PCL) and D. non-treated random (R-PCL) nanofibers.

SEM showed the cell morphology and guidance effect of aligned and random electrospun fibers. As shown in Figure 4, cells were well adhered to the surface of nanofibers with normal morphology which indicated good biocompatibility of the PCL scaffolds. Cells that colonized on the aligned nanofibrous scaffolds were oriented along the direction of the fibers in a longitudinal and bipolar fashion compared to random nanofibers (Fig 4A, B). The embryonic stem cell colonies on random oriented fibers were distributed as multi-polar forms and oriented in different directions of random nanofibers. A similar morphology was observed for colonies cultured on TCPs (Fig 4C, D). As shown in figure 4 (A, B), OD of the cell colonies on the aligned nanofibrous scaffolds showed a larger colony size with increased spreading compared to random nanofibrous scaffolds.

## Discussion

Stem cells appear to be ideal candidates for tissue regeneration (19). After culturing the cells on the scaffolds *in vitro*, implantation *in vivo* is performed, with subsequent scaffold degradation with the intent to rebuild new tissue that is complementary with the host (20).

Synthetic polymers such as poly lactic acid (PLA), PCL, poly glycolic acid (PGA), polydioxanone or poly lactide-co-glycolide (PLGA) display the ability to promote cell attachment and proliferation (21).

Studies have demonstrated that topographical cues which include a complex network of fibers, pores, ridges and other features of nanometer sized dimensions generated by the ECM may influence cell behaviors such as activation, orientation, and movement of these cells (22, 23). No study has compared the orientation effects of nanofibrous PCL on proliferation and growth of mESCs. To understand these interactions, researchers propose the use of a logical substrate which has been fabricated in the same scale as natural ECM substratum. However the difficulty with penetration of the cells in nanofibrous scaffolds that have been produced by electrospinning is a problem. This can be alleviated by plasma modification since wettability is a prerequisite for a suitable substance recognized by cells (7).

The combination of synthetic and natural polymers is one of the methods used to enhance surface hydrophilicity (24). For example, blending of PCL and poly (methacrylic acid) as a hydrophilic polymer has shown better cell attachment (25).

In this study, we used the usual plasma modification method (26) to improve hydrophilicity by oxidation of the upper atomic layer of the polymer which rendered oxygen-containing groups (-OH-COOH, etc.) on the surface of the polymer.

According to the results of this study, after plasma treatment the PCL nanofibrous scaffolds showed rapid penetration of water drops into the scaffolds. The water contact angle of the sample surfaces varied from 133 and 134.5 degrees (hydrophobicity) to approximately 0 degrees (super-hydrophilic).

SEM and the MTT assay have shown that both plasma treated aligned and random nanofibrous PCL scaffolds improved cell adhesion compared to nontreated nanofibers on day 1. Cells have a negatively charged surface due to glycol proteins and glycolipids on the plasma membrane. Plasma modifications change the neutral/hydrophobic polymer to a polar/ hydrophilic surface, therefore it can affect the interaction between cells and the upper layer of surface scaffolds (27). It has been observed that the hydrophilicity of p-PCL decreases their tensile stress.

Our results confirmed the results of a study by Ndreu et al. (28) who observed better growth and expansion of osteosarcoma cells on the modified surface of microbial polyester, (poly (3-hydroxy butyrate- co- 3-hydroxy valerate) [P(HB-co-HV)], using O_2_ plasma treatment.

Plasma treatment can affect the hydrophilic property of the scaffolds and increase cell adhesion and proliferation. However, according to our results, one day after cell seeding this increase was less than observed with TCP. This finding was consistent with the moderate porosity of scaffolds achieved at a range of 70-77% which could cause lower penetration of the cells in nanofibrous scaffolds compared to better adhesion of the cells on gelatin coated surfaces. Our results have shown that gelatin as a natural ECM protein has a better cell adhesion capacity than the plasma treated nanofibrous groups. In accordance with our study, Ghasemi-Mobarakeh et al. have shown that Schwann cells have significantly higher proliferation on gelatin/PCL scaffolds compared to PCL nanofibers (29). According to a study of adhesion mouse fibroblasts on the surface of PLGA, the adhesion property of oxygen plasma treatment combined with anchorage of cationized gelatin was more than the separate properties of gelatin and plasma (30).

Soft biodegradable materials that possess a low Young’s modulus are favorable scaffolds for regeneration of soft tissues such as internal organs, blood vessels, and nerves.

In the current study Ap-PCL had a Young’s modulus,as an indicator of elasticity, of 18.06 MPa and the Rp-PCL scaffolds had a Young’s modulus of 2.9 MPa. For A-PCL, Young’s modulus was 18.27 and for R-PCL, it was 3.16 MPa. According to the direction and thickness of nanofibers that affect tensile property, our study showed that treatment with plasma did not significantly change the tensile properties of the nanofibrous scaffold compared to the non-treated scaffolds. The mechanical properties of aligned and random oriented PCL exhibited ease of handling and good suitability for tissue regeneration with the capacity to tolerate incoming pressure. The results showed that aligned nanofibrous scaffolds had a tensile strain of 20-30%, which agreed with the range reported in the literature (11.0% ~ 40.5%) for in situ tensile strain of rabbit tibial nerves (31).

The nano-structure of PCL scaffolds that were in the range of 250-267 nm demonstrated effective induction of adherence and proliferation of mESCs. Possibly, fibers with a smaller diameter had a larger surface area which would facilitate fibers binding more growth factors and cytokines from the serum in the culture medium thereby enabling close contact and interaction between the cells and growth factors. Wang et al. studied a culture of human embryonic stem cell-derived neural progenitors on Tussah silk fibroin (TSF)-scaffold of different diameters (400 and 800 nm) and observed that on the aligned 400 nm fibers there was greater cell viability compared to those that aligned on the 800 nm fibers (32). During electrospinning the fibers lay loosely upon each other and form interconnected pores (33).

In the current study there was a slight difference on cell adhesion and proliferation in Rp-PCL compared to Ap-PCL according to the MTT test results for day 1. Although not significant, a possible explanation might be the higher porosity of random nanofibrous scaffolds. Random fibers contain numerous interconnected pores and rough surfaces that assist with additional adhesion and cell proliferation. However the smaller pore size and compact fiber arrangement in aligned electrospun nanofibrous prevents deep cell infiltration and the cells appear to spread superficially on the surface. The aligned nanofibrous scaffolds had increased density of the parallel fibers compared to random nanofibers, hence this property could limit cell entry at the time of adherence (34). Similar results have been reported by Gupta for the culture of Schwann cells on random PCL scaffold (7).

Despite the better adhesion properties of gelatin it seems that the three-dimensional substrate and its direction showed better induction in cell proliferation. We observed the highest level of cell proliferation in plasma treated aligned nanofibrous scaffolds on day 3 compared with TCP and random nanofibers. Our results were consistent with the findings of a study conducted by Engelhardt et al. who observed significantly improved adhesion and proliferation rates of smooth muscle cells (SMCs) of human coronary artery on aligned nanofibrous poly (lactic acid-co-caprolactone) [P(LA-CL)] scaffolds (35).

A possible explanation might be the more flexible and elastic nature of nanofibers compared to TCP. Christopherson et al. stated that increased cell proliferation on smaller diameter fibers was related to actin filament formation and enhanced cell spreading (36).

Similar results were observed in a study about the proliferation of Schwann cells on p-PCL nanofibrous scaffolds. Given that collagen is also one of the components of ECM proteins, the proliferation of Schwann cells on plasma treated nanofibrous scaffolds has shown a 17% increase compared with nanofibrous PCL/collagen after 8 days (26). According to these results, possibly the aligned direction of the nanofibrous scaffold affected functional changes such as cell proliferation and immigration more than some components of the ECM proteins.

As shown in MTT results, although not a significant difference, the proliferation rate between random and aligned nanofibers on day 3 showed more cell proliferation on aligned orientated scaffolds compared to random nanofibers. These results were consistent with the SEM results where cells had increased spreading on aligned nanofibers than the random nanofibers. The "contact guidance" theory which illustrates the maximum probability of the cells to migrate in directions of mechanical property of the substratum might explain this finding (32).

Possibly the proliferation and growth on aligned oriented scaffold would require less energy and time to migrate along fibers with one orientation which would provide a good opportunity for cells to exist in a larger colony size with more cell stretching compared to random nanofibers. The change in cell direction and crossing multiple different courses of random nanofibers decreases the speed of proliferation compared to aligned nanofibers, which leads to the formation of smaller colony size and less cell elongation with multipolar shape.

On day 5 we observed the highest proliferation rate in the Rp-PCL scaffolds. Supposing the highest proliferation rate (logarithmic phase) of the mESCs occurred 3 days after initial culture, the decrease in cellular proliferation in aligned nanofibrous scaffolds on day 5 could be related to the lack of space for proliferation in plasma treated aligned nanofibers (Ap-PCL) in the 96-well plate compared to random nanofibers and possibly they enter into the stationary phase of the cell cycle on day 5.

## Conclusion

In this study, we analyzed the effect of nanofiber orientation before and after plasma treatment of scaffolds on adherence, survival and proliferation of mESCs. The results have shown that plasma treatment improves the hydrophilic property of surface scaffolds providing a satisfactory adhesive substrate for cell survival and attachment compared with non-treated scaffolds. Aligned PCL scaffolds significantly promoted cell proliferation and highly supported dramatic contact guidance compared to random nanofibers. Thus the viability and proliferation of the cells were influenced by various parameters of nanofibrous scaffolds such as fiber diameter, orientation, and pores, among others.

More intensive studies are necessary to understand the mechanisms of how the topographical features of electrospun fibers affect cell growth and behavior.
